# Assessing Corpus Callosum Changes in Alzheimer's Disease: Comparison between Tract-Based Spatial Statistics and Atlas-Based Tractography

**DOI:** 10.1371/journal.pone.0035856

**Published:** 2012-04-24

**Authors:** Maria Giulia Preti, Francesca Baglio, Maria Marcella Laganà, Ludovica Griffanti, Raffaello Nemni, Mario Clerici, Marco Bozzali, Giuseppe Baselli

**Affiliations:** 1 Magnetic Resonance Laboratory, Don Gnocchi Foundation, Milan, Italy; 2 Bioengineering Department, Politecnico di Milano, Milan, Italy; 3 Neurological Rehabilitation, Don Gnocchi Foundation, University of Milan, Milan, Italy; 4 Department of Biomedical Sciences and Technologies, Don Gnocchi Foundation, University of Milan, Milan, Italy; 5 Neuroimaging Laboratory, Santa Lucia Foundation IRCCS, Rome, Italy; Hangzhou Normal University, China

## Abstract

Tractography based on Diffusion Tensor Imaging (DTI) represents a valuable tool for investigating brain white matter (WM) microstructure, allowing the computation of damage-related diffusion parameters such as Fractional Anisotropy (FA) in specific WM tracts. This technique appears relevant in the study of pathologies in which brain disconnection plays a major role, such as, for instance, Alzheimer's Disease (AD). Previous DTI studies have reported inconsistent results in defining WM abnormalities in AD and in its prodromal stage (i.e., amnestic Mild Cognitive Impairment; aMCI), especially when investigating the corpus callosum (CC). A reason for these inconsistencies is the use of different processing techniques, which may strongly influence the results. The aim of the current study was to compare a novel atlas-based tractography approach, that sub-divides the CC in eight portions, with Tract-Based Spatial Statistics (TBSS) when used to detect specific patterns of CC FA in AD at different clinical stages. FA data were obtained from 76 subjects (37 with mild AD, 19 with aMCI and 20 elderly healthy controls, HC) and analyzed using both methods. Consistent results were obtained for the two methods, concerning the comparisons AD vs. HC (significantly reduced FA in the whole CC of AD patients) and AD vs. aMCI (significantly reduced FA in the frontal portions of the CC in AD patients), thus identifying a relative preservation of the frontal CC regions in aMCI patients compared to AD. Conversely, the atlas-based method but not the TBSS showed the ability to detect a selective FA change in the CC parietal, left temporal and occipital regions of aMCI patients compared to HC. This finding indicates that an analysis including a higher number of voxels (with no restriction to tract skeletons) may detect characteristic pattern of FA in the CC of patients with preclinical AD, when brain atrophy is still modest.

## Introduction

Tractography based on Diffusion Tensor Imaging (DTI) represents a powerful tool, allowing the investigation of white matter (WM) integrity in the human brain *in vivo*, through the reconstruction of 3D bundle trajectories. Several previous studies have used DTI and tractography to assess WM damage in patients with Alzheimer's Disease (AD) and amnestic Mild Cognitive Impairment (aMCI), a clinical condition widely considered as a prodromal stage of AD [1]. Abnormalities in the architecture and microstructure of WM fibers, in fact, have been demonstrated in both these conditions (AD and aMCI), besides the well-known gray matter (GM) atrophy [2], [3]. DTI appears therefore useful in this case for the assessment of WM integrity, investigated through the observation of water molecule diffusion anisotropy [4]. This characteristic is commonly evaluated by means of the computation of Fractional Anisotropy (FA), an index derived from the tensor eigenvalues, that quantifies the diffusion directionality of water molecules [5].

However, previous DTI works have reported controversial findings in assessing specific patterns of WM damage in AD (especially at the early/moderate stages) and aMCI.

No significant changes in the corpus callosum (CC) have been reported by different studies [6]–[9] when AD patients are compared to healthy controls (HC), whereas several authors found an FA decrease in the posterior regions [10]–[14], or in the anterior ones [10], [15]. Xie et al. [16] found instead a lower FA in the genu and anterior body of the CC. Similarly, the results appear contrasting when comparing the aMCI group to the HC. The main finding was a decreased FA in the splenium of the CC [17]–[19], whereas Wang and colleagues [20] reported a decreased FA both in the genu and in the splenium. No significant changes have been found, instead, by Liu et al. [21], Bosch et al. [22], Damoiseaux et al. [23], Di Paola et al. [24]. This inconsistency in results may be partially explained by heterogeneity in patients' recruitment (individuals at different transitional stages between MCI and dementia). However, most variability across studies is also likely to be due to the different techniques used for the image analysis [21]. In previous literature, there are DTI studies on MCI and AD patients based on Region of Interest (ROI) analysis [6], [8], [12]–[14], [17]–[20], [25]–[29], tractography [30]–[33], atlas-based tractography [34] and, more recently, Tract-Based Spatial Statistics (TBSS) [3], [21]–[23], [35]–[37]. FA computation in WM structures using ROI-based approaches consists in evaluating FA values within ROIs defined *a priori*. This means that such an approach is strongly operator-dependent, time consuming, and scarcely reproducible [21]. In order to assess tract-specific FA values, tractography represents an effective technique, although diffusion abnormalities in patients' brains often compromise a successful reconstruction of individual tracts. A possible solution to this problem is provided by the use of tractographic atlases, i.e. reference patterns of tracts obtained from the average of a group of HC. This approach does not require a tract reconstruction at an individual level, and allows therefore the assessment of diffusion parameters even in pathological brains. When using a tractographic atlas, a critical issue is represented by alignment errors within the control group, or between the atlas and diseased brains. Additionally, the inter-subject variability can also produce misleading results in the application to patients. For instance, the presence of brain atrophy may cause border and partial volume effects, thus increasing the risk of including Cerebrospinal Fluid (CSF) or GM voxels in WM statistics. To reduce such a risk, the novel voxel-wise approach of TBSS was recently introduced [38], restricting the evaluation of diffusion parameters to a WM skeleton common to all the studied subjects. The FA values of each subject included in the study are projected in the same skeleton positions, allowing an analysis which does not require an *a priori* classification of individual tracts. This technique has proven the ability of increasing the objectivity, interpretability and sensitivity of multi-subject diffusion data analyses [38], and of alleviating problems related to image alignment and brain atrophy [21]. Compared to TBSS, atlas-based tractography may suffer from errors dependent on registration accuracy; nonetheless, it has the advantage of extending the analysis to the entire tract volumes, and not only to the central skeleton line. This allows a more comprehensive evaluation of WM tracts, which may be particularly relevant in the presence of a non homogeneous damage. Moreover, border effects in the atlas application can be at least partially controlled by a proper erosion of tract volumes, excluding those voxels with FA values under a certain threshold, as suggested by Reich and colleagues [39], and using a probabilistic weighing from the reference atlas. In addition, the use of a tractographic atlas allows a direct localization of the damage within a determined WM bundle and therefore a simpler and more intuitive interpretation of the results with respect to TBSS.

The aim of the present study was to compare to each other, a probabilistic atlas technique and the TBSS for the investigation of the CC in patients with AD at different stages. For this purpose, we collected DTI data from a population of patients with AD and aMCI, and from a control group of healthy elderly individuals. We employed the two techniques (probabilistic atlas technique and TBSS) to analyze the same dataset, and we highlighted advantages and disadvantages of the two approaches.

## Results

**Figure 1:**
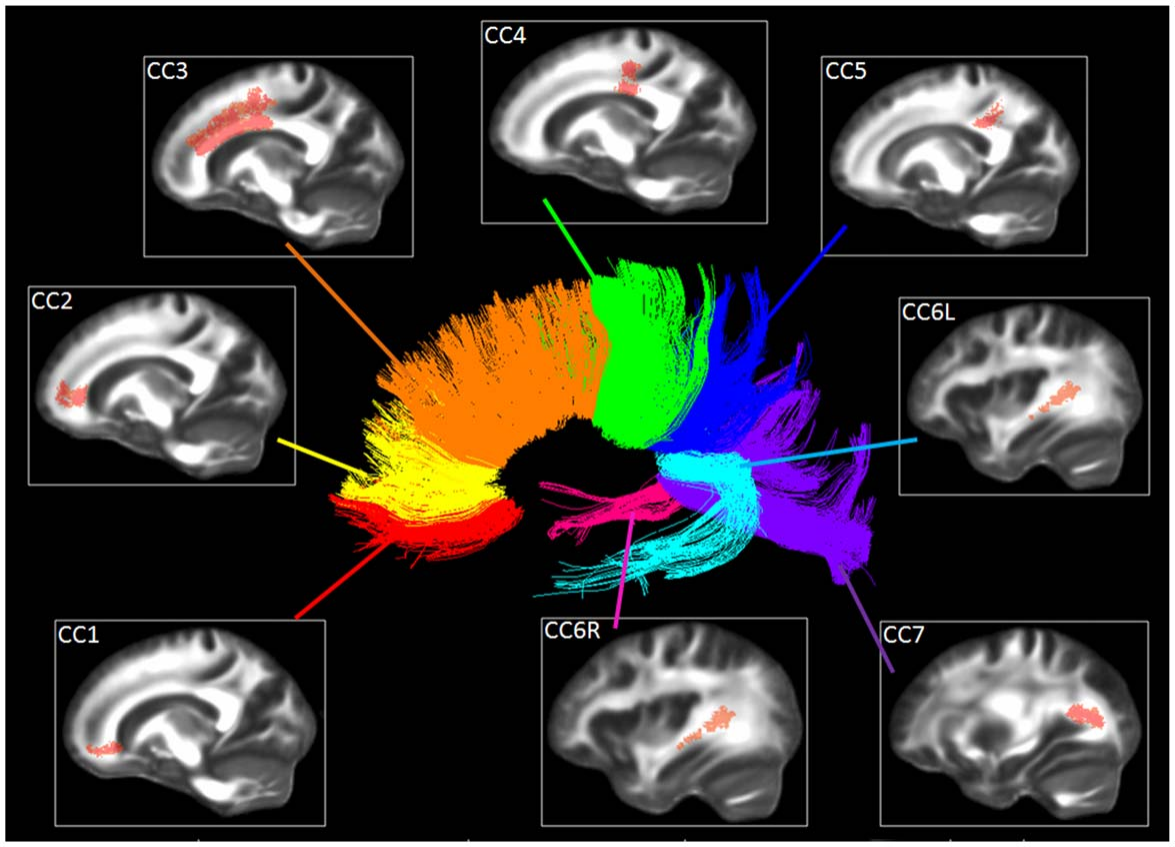
The probabilistic atlas of the CC divided in eight portions. CC1: orbital frontal, CC2: anterior frontal, CC3: superior frontal, CC4: superior parietal, CC5: posterior parietal, CC6L: left temporal, CC6R: right temporal, CC7: occipital. In the center, the CC tractographic reconstruction of one healthy subject, to better show the location of the eight portions.

**Figure 2:**
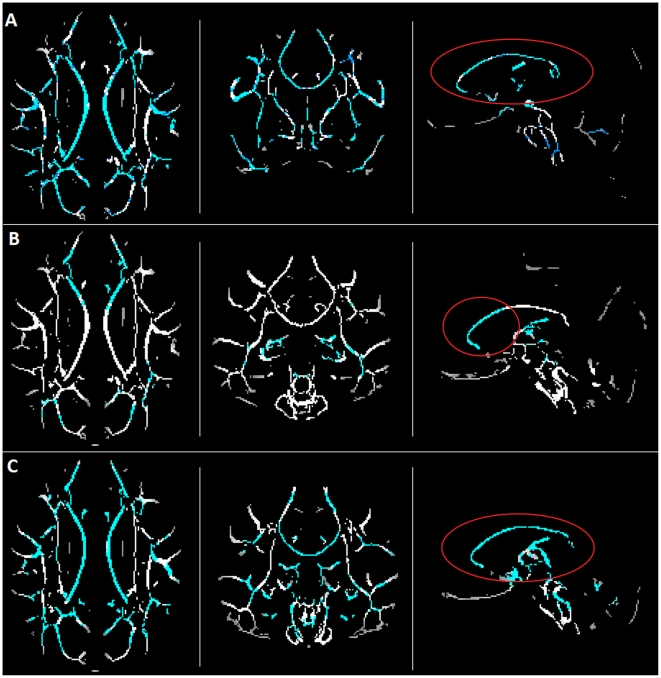
Results of the TBSS analysis. In blue, voxels with p_corr_<0.05 are highlighted. a) Comparison HC vs. aMCI; b) Comparison AD vs. aMCI; c) Comparison HC vs. AD. As highlighted by the red circles, in the comparisons a) and c) the FA of the whole CC resulted significantly reduced in AD and aMCI with respect to HC. In case b), instead, FA results significantly reduced in AD compared to aMCI only in the frontal CC regions (CC1–CC2–CC3).

The demographic data of the study sample are reported in Table 1. The probabilistic atlas of the CC divided in eight portions (orbital frontal, anterior frontal, superior frontal, superior parietal, posterior parietal, right and left temporal, occipital) is shown in Fig. 1. The results reported by the atlas-based analysis agreed with those of the TBSS regarding the comparisons of AD vs. HC and AD vs. aMCI. With both methods, in fact, significantly reduced FA values were found in all CC portions of AD patients compared to HC (p_corr_ always <0.001 with atlas-based method, see Table 2, p_corr_<0.05 with TBSS, see fig. 2C), and in the frontal CC regions (CC1-CC2-CC3) of AD compared to aMCI patients (p_corr_ always <0.013 with atlas-based method, see Table 2; p_corr_<0.05 with TBSS, see fig. 2B). Conversely, in the comparison between aMCI patients and HC, the TBSS and the atlas-based approach provided different results. In fact, the TBSS detected voxels with a statistically significant FA reduction in every CC portion of aMCI patients compared to HC (p_corr_<0.05, see fig. 2A). Conversely, the atlas-based analysis revealed a more restricted pattern of reduced FA, anatomically located in the superior frontal, parietal, occipital and left temporal CC regions (CC3-CC4-CC5-CC6L-CC7) of aMCI compared to HC (p_corr_ always <0.022, see Table 2).

**Table 1:** Demographic information of the sample.

	AD (n = 37)	aMCI (n = 19)	HC (n = 20)
**Age** (years; mean ± SD)	75.6±5.1	73.2±5.3	72±5.3
**Level of education** (years; mean ± SD)	8.2±3.7	10.2±3.6	9.1±3.8
**Sex** (M∶F)	17∶20	11∶8	8∶12
**MMSE score** (mean ± SD)	21±3.0^a^	27.2±1.4^b^	28.7±1.0^b^
**CDR** (range)	1–1.5	0–0.5	0

Demographic information and neuropsychological tests' scores. Chi square was used for gender comparison. One-way ANOVA test with Bonferroni correction for multiple comparisons was used for age, education-year, and MMSE score comparisons (significance level: : p_corr_<0.05).

a : Significant compared to aMCI and control groups;

b : Significant compared to AD group.

**Table 2:** Results of the atlas-based analysis.

Mean FA (SD)	AD	aMCI	HC	Comparison between groups (p-value)		
				AD-HC	aMCI-HC	aMCI-AD
**CC1**	0.42 (0.03)	0.44 (0.03)	0.47 (0.02)	<0.001	n.s.	0.001
**CC2**	0.49 (0.04)	0.52 (0.04)	0.55 (0.02)	<0.001	n.s.	0.004
**CC3**	0.44 (0.03)	0.46 (0.03)	0.49 (0.02)	<0.001	0.016	0.013
**CC4**	0.44 (0.03)	0.44 (0.04)	0.48 (0.03)	<0.001	0.001	n.s.
**CC5**	0.53 (0.03)	0.53 (0.05)	0.58 (0.02)	<0.001	0.004	n.s.
**CC6 L**	0.68 (0.04)	0.69 (0.05)	0.73 (0.01)	<0.001	0.022	n.s.
**CC6 R**	0.59 (0.04)	0.61 (0.04)	0.63 (0.02)	<0.001	n.s.	n.s.
**CC7**	0.54 (0.04)	0.55 (0.04)	0.60 (0.02)	<0.001	0.009	n.s.

Comparison between mean FA in the eight CC portions of the three groups of participants (groups 1–3), computed with atlas-based tractography. p-values refer to ANOVA test with correction for multiple comparisons, significance level: p_corr_<0.05.

The FA group comparisons of the skeletonized CC using an ANOVA (see Table 3) provided consistent results with the voxel-wise analysis of TBSS. In fact, the two analyses gave the same results in all group comparisons, except for the CC6R portion, where the mean FA resulted not significantly different between aMCI and HC.

**Table 3:** Results of the skeleton-based analysis of the CC.

Mean FA (SD)	AD	aMCI	HC	Comparison between groups (p-value)		
				AD-HC	aMCI-HC	aMCI-AD
**CC1**	0.52 (0.05)	0.56 (0.04)	0.60 (0.03)	<0.001	0.019	0.001
**CC2**	0.59 (0.05)	0.62 (0.04)	0.65 (0.03)	<0.001	0.026	0.006
**CC3**	0.55 (0.05)	0.58 (0.04)	0.62 (0.03)	<0.001	0.01	0.007
**CC4**	0.55 (0.04)	0.56 (0.04)	0.60 (0.03)	<0.001	0.001	n.s.
**CC5**	0.63 (0.04)	0.64 (0.04)	0.67 (0.03)	<0.001	0.007	n.s.
**CC6 L**	0.73 (0.04)	0.73 (0.05)	0.77 (0.01)	0.002	0.01	n.s.
**CC6 R**	0.61 (0.04)	0.63 (0.04)	0.64 (0.02)	<0.001	n.s.	n.s.
**CC7**	0.65 (0.05)	0.67 (0.05)	0.71 (0.02)	<0.001	0.005	n.s.

Comparison between mean FA computed in the 8 CC skeleton portions of the three groups of participants (groups 1–3). p-values refer to ANOVA test with correction for multiple comparisons, significance level: p_corr_<0.05.

The overall percentage of cases in which the atlas-based CC FA values allowed to predict the group belonging correctly (see Table 4) was 0.82 in patients with aMCI (sensitivity: 0.79; specificity: 0.85) and 0.90 in those with AD (sensitivity: 0.95; specificity: 0.80) with respect to HC, 0.82 in the recognition of AD with respect to aMCI (sensitivity: 0.89; specificity: 0.68). In contrast, the overall percentage of cases in which the TBSS-derived FA values (see Table 4) were able to identify the two clinical conditions correctly with respect to HC, was 0.74 and 0.88 for aMCI (sensitivity: 0.74; specificity: 0.75) and AD patients (sensitivity: 0.95; specificity: 0.75) respectively, whereas the detection of AD with respect to aMCI succeeded in an overall percentage of 0.79 (sensitivity: 0.84; specificity: 0.68). The generalizability of these findings is supported by a 80/20% cross-validation (see Table 5).

**Table 4:** Comparison between atlas-based tractography and TBSS in terms of overall percentage of correct pathology detection, sensitivity and specificity.

Pathology	Method	Overall %	Sensitivity	Specificity
**AD detection (over HC)**	*Atlas-based*	0.90	0.95	0.80
	*TBSS*	0.88	0.95	0.75
**aMCI detection (over HC)**	*Atlas-based*	0.82	0.79	0.85
	*TBSS*	0.74	0.74	0.75
**AD detection (over aMCI)**	*Atlas-based*	0.82	0.89	0.68
	*TBSS*	0.79	0.84	0.68

Overall percentage of correct pathology detection, sensitivity and specificity of the two experimented techniques in the detection of AD and aMCI from healthy controls and in the detection of AD from aMCI.

**Table 5:** 80/20% Cross-validation of the logistic regression model.

Pathology	Method	Overall % - Selected cases	Overall % - Unselected cases
**AD detection (over HC)**	*Atlas-based*	0.89	0.91
	*TBSS*	0.86	0.87
**aMCI detection (over HC)**	*Atlas-based*	0.81	0.75
	*TBSS*	0.74	0.75
**AD detection (over aMCI)**	*Atlas-based*	0.85	0.78
	*TBSS*	0.79	0.78

The overall percentage of correct pathology detection for the validation sample (unselected cases) was always no more than 10% lower than the accuracy rate for the training sample (selected cases), suggesting that the logistic regression model based on this analysis would be effective also applied to cases not included in the sample.

## Discussion

The present study aimed at comparing the performance of an atlas-based tractography approach in the assessment of CC damages in aMCI and mild AD, with the widely accepted approach of TBSS. We didn't consider all the other issues of the DTI processing already studied in literature (i.e. different tractography thresholds, different diffusion weighted image alignment and DT estimation methods) that could lead to different clinical findings, but we focused on two different approaches of FA estimation in specific WM regions, starting from the same FA maps. When comparing AD patients with HC and aMCI, the results of the atlas-based approach were strongly consistent with those obtained by TBSS, thus supporting the reliability of the former method. This is also confirmed by the high sensitivity (always >0.79) and specificity (always >0.80) shown by the atlas-based method in the correct classification of AD and aMCI patients from HC (see Table 4).

From a physiopathological viewpoint, these results confirm that different patterns of WM abnormalities in the CC, are associated to different stages of AD. This supports the hypothesis that brain disconnection plays a critical role in AD pathophysiology, and contributes to the progressive accumulation of cognitive disabilities in the transitional stage between normal aging and dementia [3], [40]–[41].

The delineation of the pattern of CC abnormalities in aMCI patients appears to be more informative when using the atlas-based analysis than the TBSS. The atlas-based method, in fact, showed the ability to identify those CC regions (CC1-CC2-CC6R) which are still preserved in aMCI patients, but become damaged at later disease stages. This more restricted pattern of CC damage in aMCI patients is consistent both with the distribution of GM loss that has been found in many volumetric studies [42]–[44], and with the neuropsychological profile of the patients [45]–[47]. The early selective damage of the central and posterior CC subregions, in fact, appears in concordance with previous main findings on aMCI [17], [19], [23] and is consistent with the pathological knowledge we have of AD progression, according to which the posterior CC subregions should be involved in the earlier stages of the disease and the anterior CC subregions only in the later stages [48], [49]. Considering that aMCI represents the earliest AD stage detectable by clinical and neuropsychological instruments, we might speculate that this posterior WM disconnection may contribute in determining the clinical onset of the disease (memory deficits responding to the criteria for a diagnosis of aMCI) [3], [40]–[41].

Moreover, the different damage of the right and left temporal fibers (CC6L>CC6R) in aMCI with respect to HC, highlighted with the atlas based method, is in concordance with functional neuroimaging findings, which suggest a higher activation of the right medial temporal lobe as a consequence of a structural deficit of the left one [50], [51]. These fibers (CC6L) are indeed originating from the left temporo-parietal GM, which is primarily affected in the early stages of AD [21], [36], [52], [53].

The higher capability of the atlas-based approach in the delineation of tissue damage in aMCI appears to be supported by the higher values of sensitivity (0.79 vs. 0.73) and specificity (0.85 vs. 0.75) obtained with this method (compared to the TBSS) in correctly classifying patients' group belonging. A possible explanation for the higher accuracy of the atlas-based approach in the definition of CC abnormalities in aMCI might be related to the different analysis performed on the single tracts by the two methods: a skeleton-based approach, as TBSS, evaluates only the central line of the tracts, thus ignoring their whole extent, which can be particularly important in widespread tracts such as the CC. On one hand, TBSS preserves in this way from misclassifying brain atrophy as microscopic tissue damage. On the other hand, this technique subtracts voxels that may be relevant for a correct assessment of certain WM tracts status in the early disease stages, when atrophy is not yet remarkable. Basically, TBSS reduces misregistration artifacts by reducing anatomical information to the WM core, and this might be a reason why TBSS and atlas-based findings appear different.

The segmentation of the CC in eight portions, instead of the common subdivision in three or four regions (rostrum, genu, body and splenium), allowed a more detailed localization of the damage [54]. To our knowledge, this is the first study that used a probabilistic atlas of the CC divided in eight portions to analyze DTI data. Further, the probabilistic atlas does not require tensor registration and reorientation steps, as in recently proposed approaches of atlas reconstruction [55], and, additionally, it provides robust information regarding the probability of a WM location to belong to a specific tract, permitting the consequent weighing and thresholding of tract-specific DTI parameters. Therefore, differently from the TBSS analysis, the application of the atlas has the advantage to allow a better anatomical interpretation of the results by focusing the analysis on voxels belonging (with a high confidence) to a certain bundle of interest. In fact, to interpret the TBSS results, the masking with the constructed CC atlas was necessary to observe selectively the results regarding the tract portion of interest. In the absence of an atlas, the localization of the damage within the WM would have been less accurate with TBSS, in terms of belonging to a specific tract.

Misregistration artifacts, which usually affect the atlas-based methods more than the TBSS, can be effectively controlled thanks to the probabilistic definition of the atlas. This indeed allows the limitation to high membership probabilities. Moreover, the exclusion of all FA values under the commonly adopted threshold of 0.2 in the atlas application, helps to avoid the erroneous inclusion in the analysis of voxels not belonging to WM.

A limit of the present comparison of atlas-based and TBSS methods could be the different statistical analysis involved in the two techniques, due to their different intrinsic nature (voxel-based or average-based). Nonetheless, the computation of the average FA in the skeleton divided by the eight CC regions, allowed us to restrict the observation of TBSS results to the CC and to perform an identical statistical analysis for between group comparisons (ANOVA model) in both methods. The results provided by this analysis, shown in Table 3, were consistent with the findings of the TBSS, proving the fairness of the direct comparison between the voxel-wise TBSS and the atlas-based method. In fact, the two methods gave the same results as regards the comparisons of AD vs. HC and AD vs. aMCI, and differ only for the mean CC6R FA in the comparison HC vs. aMCI, which appears non significantly different between the two groups. This could find an explanation in the fact that the voxels detected by TBSS as significantly different are a few in the CC6R section and the significance of the difference disappear when performing the average. In all the other comparisons, though, the results of the average-based analysis on the skeleton portions reflect the ones of TBSS.

In conclusion, this study shows the reliability of an atlas-based method, based on the use of a probabilistic atlas of the CC divided in eight portions, which allows an accurate analysis of the WM tracts in their entire extent. The performances of the two experimented techniques (atlas based approach and TBSS) used for FA analysis in the CC appear similar when comparing AD vs. aMCI and HC, but different when comparing aMCI to HC. In the latter case, the atlas-based tractography proved to be more sensitive in delineating the pattern of patients' CC damage.

## Materials and Methods

### Subjects

Diffusion weighted images were obtained from 76 participants (see Table 1 for detailed clinical information), divided in four groups: 1) 19 patients (age 73.2±5.3; 11 males) diagnosed with aMCI according to Petersen criteria [1] and to Grundman and colleagues operational criteria [56]: memory complaint, confirmed by an informant; abnormal memory function, documented by previous extensive neuropsychological evaluation; normal general cognitive function, as determined by both Clinical Dementia Rating (CDR [57]) scale (CDR with at least a 0.5 in the memory domain) and Mini-Mental State Examination (MMSE [58]) score (MMSE greater than or equal to 24); no impairment in functional activities of daily living as determined by a clinical interview with the patient and informant; no significant cerebral vascular disease (Hachinski score less than or equal to 4 [59]); no major psychiatric illnesses with particular attention to exclude subjects with history of depression (Hamilton Depression Rating Scale score less than or equal to 12 [60]); 2) 37 patients (age 75.6±5.1, 17 males) meeting the diagnosis of probable AD according to the NINCDS-ADRDA criteria [61] and to the updated guidelines for AD of the National Institute on Aging Alzheimer's Association [62]; all AD patients were in mild to moderate stage of the disease according to CDR scale (0.5 to 2) and to MMSE score (between 18 and 24); 3) 20 HC (age 72.0±5.3; 8 males), used for between group-comparisons; 4) additional 25 HC used only for atlas construction purpose (age 70.2±5.1, 11 males). No significant differences were found in age and gender between all groups. All patients were recruited from the specialist dementia clinic of the Fondazione Don Carlo Gnocchi, Milan, Italy. HC were preliminarily screened to exclude major systemic, psychiatric or neurological illnesses. The study was conformed to the ethical principles of the Helsinki Declaration and was approved by the Don Carlo Gnocchi Foundation ethical committee. Informed written consent was obtained from all subjects before study initiation. Patients' T2-weigthed scans were reviewed by an experienced neurologist to exclude the presence of WM hyperintensities outside the normal range.

### Magnetic Resonance Acquisitions

Brain Magnetic Resonance acquisitions were performed using a 1.5 Tesla scanner (Siemens Magnetom Avanto, Erlangen, Germany), including the following sequences: 1) dual-echo turbo spin echo (TR = 2650 ms, TE = 28/113 ms, echo train length = 5, flip angle = 150°, 50 interleaved 2.5-mm-thick axial slices, matrix size = 256×256 interpolated to 512×512, FOV = 250 mm×250 mm); 2) diffusion weighted pulsed-gradient spin-echo planar (TR = 7000 ms, TE = 94 ms, 50 2.5-mm-thick axial slices, matrix size = 128×96, FOV = 320 mm×240 mm), with diffusion gradients (b-value = 900 s/mm2) applied in 12 non-collinear directions. Two runs of images were acquired for each subject, each one including diffusion weighted images and one b = 0 image (without diffusion weighting).

### DTI Analysis

Diffusion weighted images were corrected for eddy current distortions using FSL (http://www.fmrib.ox.ac.uk/fsl/). Brain was extracted using the FSL Brain Extraction Tool (BET). For every subject, the two runs were registered to the same stereotaxic space using SPM5 (www.fil.ion.ucl.ac.uk/spm), by estimating the transformation parameters between the b = 0 image of the second run and the b = 0 image of the first run, and by applying them to all the DW images of the second run. The diffusion tensor was estimated by using Diffusion Toolkit (www.trackvis.org) v0.6, which first rotates the B-matrix for slice angulation and for the rotation applied by FSL and SPM. The tensor was then diagonalized, obtaining its eigenvalues. Finally, the tensor scalar invariant FA was computed for each subject.

### CC probabilistic atlas construction

For every healthy subject selected to build up the atlas (group 4), tractography was performed by Diffusion Toolkit v0.6, using the brute force approach and the Interpolated Streamline deterministic algorithm. An angle of 35° and an FA threshold of 0.2 were adopted as stopping criteria. For each subject, the CC was segmented in eight portions, by following a subdivision in seven portions suggested in previously published guidelines [54] and further dividing the temporal section in left and right fibers. The ROIs of way-points were manually positioned on the FA maps using Trackvis (www.trackvis.org) v0.5.1, and density maps of the reconstructed tracts were created. Then, FA images were non-linearly registered to the MNI152 standard space with SPM5, using the FMRIB58_FA template available in FSL as reference image for the alignment. For each HC, the estimated transformation between his/her FA map and the template was applied to the correspondent tract density maps of the eight segmented CC portions. The tract density maps were then binarised and averaged separately for each CC portion, in order to obtain images indicating the probability of each voxel to belong to the considered tract portion. With the aim of increasing the confidence of belonging to the tract of interest, probability maps were thresholded above 90% probability (the probability under 90% was set to zero, whereas the probability over 90% preserved the original value).

### CC FA analysis using atlas-based tractography

Average FA values along the tracts in the eight CC portions were extracted for every subject using the following atlas-based method. First of all, the FA maps of all subjects were non-linearly registered to the atlas space (in MNI coordinates) using FSL FNIRT and the FMRIB58_FA template available in FSL as reference image. For every CC portion (as defined on the atlas), mean FA values were derived, for each subject, using an in-house made Matlab script. This script first masked the registered FA maps from all subjects used for between group comparisons (groups 1–3) with the constructed atlas. Then, for each CC atlas portion and for each subject, it computed the average FA weighted for the probability of every voxel to belong to the considered CC portion. In order to minimize the probability of including CSF or GM, those voxels with FA values <0.2 were rejected from the analysis [39]. Between-group statistics was performed using SPSS Statistics v17.0 (www.spss.com). An ANOVA model was employed to test for between group differences in mean FA for each considered portion of the CC. Bonferroni's correction was used to account for multiple comparisons (p<0.05). The overall percentage of cases in which the CC FA mean values of the different CC sections allowed to correctly predict patients' condition (aMCI or AD) was determined with a logistic regression model implemented using SPSS v17.0. Sensitivity and specificity of the model were also computed. A 80/20% cross-validation was performed in SPSS in order to verify the effectiveness of the regression model.

### CC FA Analysis using TBSS

The TBSS v1.2 [38], part of FSL was performed. All subjects' FA maps were nonlinearly aligned to a 1×1×1 mm standard space in MNI152 coordinates, using FSL FNIRT and FMRIB58_FA as template image (same registration of the atlas-based method). A template skeleton derived from the FMRIB58_FA was used for the analysis. This skeleton was thresholded at a value of 0.2 and, for every subject, individual FA data were projected into it, as described in [38]. Following the standard TBSS procedure, data were then fed into voxelwise statistics to test for the following group comparisons: 1) HC vs. aMCI; 2) HC vs. AD; 3) aMCI vs. AD. The permutation tool “randomize” was used, by setting 5000 permutations and a statistical threshold of p<0.05. The Threshold-Free Cluster-Enhancement (TFCE) was adopted as correction for multiple comparisons. The statistical maps containing those voxels with significantly different FA values between groups, were masked with the eight portions of the CC identified on the atlas, in order to observe significant different voxels in each CC portion separately. To verify the reliability of the comparison between the atlas-based technique and the TBSS, which involved two different statistics (voxelwise for the TBSS, difference between averages for atlas-based method) due to the intrinsic nature of the two methods, we performed an additional analysis on TBSS data, by extracting the average FA's for each subject on the skeletons found in each CC atlas region and by exploring the between group FA differences with an ANOVA model, exactly as for the atlas-based method. The overall percentage of cases in which the skeleton CC FA values allowed to correctly detect patients' condition (aMCI or AD) was determined with a logistic regression model implemented using SPSS v17.0. Sensitivity and specificity of the model were also computed. Moreover, a 80/20% cross-validation was performed in SPSS in order to verify the effectiveness of the regression model.

## References

[1] Petersen RC (2004). Mild cognitive impairment as a diagnostic entity.. J Intern Med.

[2] Di Paola M, Spalletta G, Caltagirone C (2010). In vivo structural neuroanatomy of corpus callosum in alzheimer's disease and mild cognitive impairment using different MRI techniques: A review.. J Alzheimers Dis.

[3] Serra L, Cercignani M, Lenzi D, Perri R, Fadda L (2010). Grey and white matter changes at different stages of alzheimer's disease.. J Alzheimers Dis.

[4] Mori S, Zhang J (2006). Principles of diffusion tensor imaging and its applications to basic neuroscience research.. Neuron.

[5] Pierpaoli C, Basser PJ (1996). Toward a quantitative assessment of diffusion anisotropy.. Magn Reson Med.

[6] Fellgiebel A, Wille P, Muller MJ, Winterer G, Scheurich A (2004). Ultrastructural hippocampal and white matter alterations in mild cognitive impairment: A diffusion tensor imaging study.. Dement Geriatr Cogn Disord.

[7] Head D, Buckner RL, Shimony JS, Williams LE, Akbudak E (2004). Differential vulnerability of anterior white matter in nondemented aging with minimal acceleration in dementia of the Alzheimer type: evidence from diffusion tensor imaging.. Cereb Cortex.

[8] Stahl R, Dietrich O, Teipel SJ, Hampel H, Reiser MF (2007). White matter damage in alzheimer disease and mild cognitive impairment: Assessment with diffusion-tensor MR imaging and parallel imaging techniques.. Radiology.

[9] Choi SJ, Lim KO, Monteiro I, Reisberg B (2005). Diffusion tensor imaging of frontal white matter microstructure in early Alzheimer's disease: a preliminary study.. J Geriatr Psychiatry Neurol.

[10] Bozzali M, Falini A, Franceschi M, Cercignani M, Zuffi M (2002). White matter damage in Alzheimer's disease assessed in vivo using diffusion tensor magnetic resonance imaging.. J Neurol Neurosurg Psychiatry.

[11] Takahashi S, Yonezawa H, Takahashi J, Kudo M, Inoue T (2002). Selective reduction of diffusion anisotropy in white matter of Alzheimer disease brains measured by 3.0 Tesla magnetic resonance imaging.. Neurosci Lett.

[12] Zhang Y, Schuff N, Jahng GH, Bayne W, Mori S (2007). Diffusion tensor imaging of cingulum fibers in mild cognitive impairment and alzheimer disease.. Neurology.

[13] Naggara O, Oppenheim C, Rieu D, Raoux N, Rodrigo S (2006). Diffusion tensor imaging in early alzheimer's disease.. Psychiatry Res.

[14] Duan JH, Wang HQ, Xu J, Lin X, Chen SQ (2006). White matter damage of patients with alzheimer's disease correlated with the decreased cognitive function.. Surg Radiol Anat.

[15] Teipel SJ, Stahl R, Dietrich O, Schoenberg SO, Perneczky R (2007). Multivariate network analysis of fiber tract integrity in Alzheimer's disease.. Neuroimage.

[16] Xie S, Xiao JX, Gong GL, Zang YF, Wang YH (2006). Voxel-based detection of white matter abnormalities in mild Alzheimer disease.. Neurology.

[17] Ukmar M, Makuc E, Onor ML, Garbin G, Trevisiol M (2008). Evaluation of white matter damage in patients with alzheimer's disease and in patients with mild cognitive impairment by using diffusion tensor imaging.. Radiol Med.

[18] Parente DB, Gasparetto EL, da Cruz LC, Domingues RC, Baptista AC (2008). Potential role of diffusion tensor MRI in the differential diagnosis of mild cognitive impairment and alzheimer's disease.. AJR Am J Roentgenol.

[19] Cho H, Yang DW, Shon YM, Kim BS, Kim YI (2008). Abnormal integrity of corticocortical tracts in mild cognitive impairment: A diffusion tensor imaging study.. J Korean Med Sci.

[20] Wang L, Goldstein FC, Veledar E, Levey AI, Lah JJ (2009). Alterations in cortical thickness and white matter integrity in mild cognitive impairment measured by whole-brain cortical thickness mapping and diffusion tensor imaging.. AJNR Am J Neuroradiol.

[21] Liu Y, Spulber G, Lehtimaki KK, Kononen M, Hallikainen I (2009). Diffusion tensor imaging and tract-based spatial statistics in alzheimer's disease and mild cognitive impairment.. Neurobiol Aging.

[22] Bosch B, Arenaza-Urquijo EM, Rami L, Sala-Llonch R, Junque C (2010). Multiple DTI index analysis in normal aging, amnestic MCI and AD. relationship with neuropsychological performance.. Neurobiol Aging.

[23] Damoiseaux JS, Smith SM, Witter MP, Sanz-Arigita EJ, Barkhof F (2009). White matter tract integrity in aging and alzheimer's disease.. Hum Brain Mapp.

[24] Di Paola M, Di Iulio F, Cherubini A, Blundo C, Casini AR (2010). When, where and how corpus callosal changes in MCI and AD: a multimodal MRI study.. Neurology.

[25] Rogalski EJ, Murphy CM, deToledo-Morrell L, Shah RC, Moseley ME (2009). Changes in parahippocampal white matter integrity in amnestic mild cognitive impairment: A diffusion tensor imaging study.. Behav Neurol.

[26] Chen TF, Lin CC, Chen YF, Liu HM, Hua MS (2009). Diffusion tensor changes in patients with amnesic mild cognitive impairment and various dementias.. Psychiatry Res.

[27] Medina D, DeToledo-Morrell L, Urresta F, Gabrieli JD, Moseley M (2006). White matter changes in mild cognitive impairment and AD: A diffusion tensor imaging study.. Neurobiol Aging.

[28] Chen SQ, Kang Z, Hu XQ, Hu B, Zou Y (2007). Diffusion tensor imaging of the brain in patients with alzheimer's disease and cerebrovascular lesions.. J Zhejiang Univ Sci B.

[29] Chua TC, Wen W, Chen X, Kochan N, Slavin MJ (2009). Diffusion tensor imaging of the posterior cingulate is a useful biomarker of mild cognitive impairment.. Am J Geriatr Psychiatry.

[30] Zhang Y, Schuff N, Du AT, Rosen HJ, Kramer JH (2009). White matter damage in frontotemporal dementia and alzheimer's disease measured by diffusion MRI.. Brain.

[31] Nakata Y, Sato N, Abe O, Shikakura S, Arima K (2008). Diffusion abnormality in posterior cingulate fiber tracts in alzheimer's disease: Tract-specific analysis.. Radiat Med.

[32] Morikawa M, Kiuchi K, Taoka T, Nagauchi K, Kichikawa K (2010). Uncinate fasciculus-correlated cognition in alzheimer's disease: A diffusion tensor imaging study by tractography.. Psychogeriatrics.

[33] Taoka T, Morikawa M, Akashi T, Miyasaka T, Nakagawa H (2009). Fractional anisotropy–threshold dependence in tract-based diffusion tensor analysis: Evaluation of the uncinate fasciculus in alzheimer disease.. AJNR Am J Neuroradiol.

[34] Pievani M, Agosta F, Pagani E, Canu E, Sala S (2010). Assessment of white matter tract damage in mild cognitive impairment and alzheimer's disease.. Hum Brain Mapp.

[35] Zhuang L, Wen W, Zhu W, Trollor J, Kochan N (2010). White matter integrity in mild cognitive impairment: A tract-based spatial statistics study.. Neuroimage.

[36] Stricker NH, Schweinsburg BC, Delano-Wood L, Wierenga CE, Bangen KJ (2009). Decreased white matter integrity in late-myelinating fiber pathways in alzheimer's disease supports retrogenesis.. Neuroimage.

[37] Haller S, Nguyen D, Rodriguez C, Emch J, Gold G (2010). Individual prediction of cognitive decline in mild cognitive impairment using support vector machine-based analysis of diffusion tensor imaging data.. J Alzheimers Dis.

[38] Smith SM, Jenkinson M, Johansen-Berg H, Rueckert D, Nichols TE (2006). Tract-based spatial statistics: Voxelwise analysis of multi-subject diffusion data.. Neuroimage.

[39] Reich DS, Ozturk A, Calabresi PA, Mori S (2010). Automated vs. conventional tractography in multiple sclerosis: Variability and correlation with disability.. Neuroimage.

[40] Bozzali M, Padovani A, Caltagirone C, Borroni B (2011). Regional grey matter loss and brain disconnection across alzheimer disease evolution.. Curr Med Chem.

[41] Gili T, Cercignani M, Serra L, Perri R, Giove F (2011). Regional brain atrophy and functional disconnection across alzheimer's disease evolution.. J Neurol Neurosurg Psychiatry.

[42] Kakeda S, Korogi Y (2010). The efficacy of a voxel-based morphometry on the analysis of imaging in schizophrenia, temporal lobe epilepsy, and alzheimer's disease/mild cognitive impairment: A review.. Neuroradiology.

[43] Karas G, Sluimer J, Goekoop R, van der Flier W, Rombouts SA (2008). Amnestic mild cognitive impairment: Structural MR imaging findings predictive of conversion to alzheimer disease.. AJNR Am J Neuroradiol.

[44] Vemuri P, Weigand SD, Knopman DS, Kantarci K, Boeve BF (2011). Time-to-event voxel-based techniques to assess regional atrophy associated with MCI risk of progression to AD.. Neuroimage.

[45] Serra L, Perri R, Cercignani M, Spano B, Fadda L (2010). Are the behavioral symptoms of alzheimer's disease directly associated with neurodegeneration?. J Alzheimers Dis.

[46] Risacher SL, Saykin AJ, West JD, Shen L, Firpi HA (2009). Baseline MRI predictors of conversion from MCI to probable AD in the ADNI cohort.. Curr Alzheimer Res.

[47] Leube DT, Weis S, Freymann K, Erb M, Jessen F (2008). Neural correlates of verbal episodic memory in patients with MCI and alzheimer's disease–a VBM study.. Int J Geriatr Psychiatry.

[48] Brun A, Englund E (2002). Regional pattern of degeneration in Alzheimer's disease: neuronal loss and histopathological grading.. Histopathology.

[49] Brun A, Englund E (1981). Regional pattern of degeneration in Alzheimer's disease: neuronal loss and histopathological grading.. Histopathology.

[50] Fleisher AS, Houston WS, Eyler LT, Frye S, Jenkins C (2005). Identification of Alzheimer disease risk by functional magnetic resonance imaging.. Arch Neurol.

[51] Dickerson BC, Salat DH, Bates JF, Atiya M, Killiany RJ (2004). Medial temporal lobe function and structure in mild cognitive impairment.. Ann Neurol.

[52] Shi F, Liu B, Zhou Y, Yu C, Jiang T (2009). Hippocampal volume and asymmetry in mild cognitive impairment and Alzheimer's disease: Meta-analyses of MRI studies.. Hippocampus.

[53] Cherbuin N, Réglade-Meslin C, Kumar R, Sachdev P, Anstey KJ (2010). Mild Cognitive Disorders are Associated with Different Patterns of Brain asymmetry than Normal Aging: The PATH through Life Study.. Front Psychiatry.

[54] Lebel C, Caverhill-Godkewitsch S, Beaulieu C (2010). Age-related regional variations of the corpus callosum identified by diffusion tensor tractography.. Neuroimage.

[55] Peng H, Orlichenko A, Dawe RJ, Agam G, Zhang S (2009). Development of a human brain diffusion tensor template.. Neuroimage.

[56] Grundman M, Petersen RC, Ferris SH, Thomas RG, Aisen PS (2004). Mild cognitive impairment can be distinguished from Alzheimer disease and normal aging for clinical trials.. Arch Neurol.

[57] Morris JC (1993). The clinical dementia rating (CDR): Current version and scoring rules.. Neurology.

[58] Magni E, Binetti G, Bianchetti A, Rozzini R, Trabucchi M (1996). Mini-mental state examination: A normative study in italian elderly population.. Eur J Neurol.

[59] Rosen WG, Terry RD, Fuld PA, Katzman R, Peck A (1980). Pathological verification of ischemic score in differentiation of dementias.. Ann Neurol.

[60] Hamilton M (1960). A rating scale for depression.. J Neurol Neurosurg Psychiatry.

[61] McKhann G, Drachman D, Folstein M, Katzman R, Price D (1984). Clinical diagnosis of alzheimer's disease: Report of the NINCDS-ADRDA work group under the auspices of department of health and human services task force on alzheimer's disease.. Neurology.

[62] McKhann GM, Knopman DS, Chertkow H, Hyman BT, Jack CR (2011). The diagnosis of dementia due to alzheimer's disease: Recommendations from the national institute on aging-alzheimer's association workgroups on diagnostic guidelines for alzheimer's disease.. Alzheimers Dement.

